# China is implementing the national nutrition plan of action

**DOI:** 10.3389/fnut.2022.983484

**Published:** 2022-08-22

**Authors:** Li Cai, Xinyi Hu, Shuang Liu, Lei Wang, Xuehong Wang, Hua Tu, Yeqing Tong

**Affiliations:** ^1^Wuhan Center for Disease Control and Prevention, Wuhan, China; ^2^School of Public Health, Wuhan University, Wuhan, China; ^3^Global Study Institute, University of Geneva, Geneva, Switzerland; ^4^Hubei Center for Disease Control and Prevention, Wuhan, China; ^5^China University of Geosciences, Wuhan, China; ^6^Wuhan Institute of Physical Education, Wuhan, China

**Keywords:** nutrition, plan, nutrition education, technology-enhanced learning, China

## Abstract

With the transformation of Chinese economic and social structure, the social determinants of nutrition have undergone significant changes, which have a great influence on the dietary patterns and nutrition status of its population. The transition in the structure of nutrition intake in China can be characterized by a rapid trend in food accessibility, affordability, and diversity. However, challenges, such as the triple burden of malnutrition (undernourishment, micronutrient deficiencies, and obesity), still seriously threaten the population's health. To approach the targets for the “Healthy China 2030,” the General Office of the State Council of the People's Republic of China issued the National Nutrition Plan of Action (2017–2030), which specifies multi-sectoral transdisciplinary measures to improve population nutrition status. In our study, we recognized the role of the National Nutrition Plan of China in responding to the call for the Sustainable Development Goals (SDGs). Under the global- and country-level targets, this paper presented six primary intervention measures specified in the National Nutrition Plan of Action in China, highlighting the importance of collaborative actions for participants in all sectors.

## Introduction

Adopted by the United Nations (UN) in 2015, the Sustainable Development Goals provided the global actors with a shared blueprint of targets to be achieved by 2030, in which all goals are interconnected. As nutrition is closely related to the realization of a set of interdisciplinary targets, such as human well-being, social equality, and economic development, the importance of updating and monitoring country-based nutrition planning has become critical (1–4).

As a transition economy, China has undergone great changes in the nutrition status of its population. In the past few decades, economic development and national health governance have improved the overall nutritional status substantially while have also brought new challenges. The 2022 Chinese Dietary Guidelines (CDG) navigated the main trends in Chinese dietary structures, which in the past decades, have shifted from plant-based or plant-forward diets to whole-food diets. Although traditional plant-based diets are rich in dietary fiber, they are often deficient in animal food, dairy products, and fruits. With the continuous transformation of dietary structure, residents in some areas consume too much meat (especially pork) and oils. A striking decline in the consumption of coarse grains can be observed. The transition may lead to imbalanced nutrient intake and an increasing incidence of some chronic diseases, such as hypertension (5–7). The high-sodium, low-whole grain, and low-fruit/nut/vegetable diet presented in the current Chinese dietary structure are factors causing cardiovascular death and disease burden in China. In 2017, about 2.6 million cardiovascular deaths in China could be attributed to diet. The disease burden of cardiovascular disease exceeds 85 million disability-adjusted life years (DALYs), of which about 56 million are attributed to unhealthy diets (8, 9).

Nutrition is one of the most essential and cost-effective factors that contribute to public health. Under the initiative of SDGs and the Healthy China 2030, a corresponding action on nutrition plans was released in 2017 by the Chinese State Council (10–12). The National Nutrition Plan (2017–2030) of Action in China encompassed efforts in six areas, which targeted six goals for 2030.

## Action targets for 2030

The action targets 2030 were formulated on the basis of mostly realization of the 2020 goals.

- Further decrease the prevalence rate of anemia in key populations. Key populations, encompassing children under the age of five (U5) and pregnant women, have an overall anemia prevalence of <10%.
- The prevalence of stunting among U5 declines to 5%. The exclusive breastfeeding (EBF) rate for infants aged 0–6 months will increases by 10% from 2020.
- Further narrow the difference in height between urban and rural students; the rising trend of student obesity can be effectively controlled.
- Further increase the coverage of nutrition screening among inpatients and the proportion of nutritional treatment for malnourished inpatients.
- The penetration rate of nutrition knowledge will increase by 10% on the basis of 2020.
- The per capita daily salt intake will reduce by 20%; residents' growing prevalence of overweight and obesity slows down significantly.

## Six major intervention actions

To achieve the Action Targets for 2030, health governors have listed six actions to initiate interdisciplinary, interdepartmental actions, not only for key populations but for all.

### Nutrition actions of the first 1,000 days

The growth and development trajectory is heterogeneous across the lifespan. Many vital functions are developed in the first 1,000 days of newborns (13). National Nutrition Plan encompassed four actions in this area:

#### Popularize nutrition screening and dietary guidance for women before pregnancy and during pregnancy

To achieve that, health facilities above the county level should provide nutrition guidelines for pregnant women. Nutrition screening and dietary guidelines should be included in the package of check-ups during pre-pregnancy and pregnancy. Efficient interventions should be implemented after screening to effectively reduce the incidence of a newborn being underweight or macrosomia. Counseling platforms specializing in the first 1,000 days nutrition should be established to provide timely answers for people needed.

#### Initiate nutrition intervention programs for women and children

Specifically, folic acid supplementation programs are advocated for rural women to prevent neural tube defects. Launching micronutrient supplementation programs for peri-pregnant women is recommended to reduce the prevalence of anemia during pregnancy and prevent neonatal malnutrition. Evidence-based nutrition packages are suggested to be delivered to pregnant women under the framework of project management.

#### Improve breastfeeding rates and further promote scientific feeding

To be specific, to build and improve breastfeeding places. Nursing rooms can be established in public places; research on the scientific feeding of infants should be supported.

#### Improve the quality and safety of infant foods

Specific measures include improving the research and development capabilities of infant food, timely revision of infant formula and complementary food standards, and monitoring the nutrient content of infant food.

### Improve nutrition status of students

Student malnutrition can attribute to undernutrition or over-nutrition. It can be caused by unhealthy and unbalanced food intake or defects in how the body digests the food (14, 15). The dietary adequacy and nutritional requirements should be updated timely during the fast-growing period of children and adolescents (16, 17).

- Provide food guidelines for students. It is encouraged to set up age-specific guidelines based on local dietary characteristics.
- Provide timely intervention for overweight and obese students. Specifically, weight management programs combining exercise and nutrition can be offered to students. It is also urgent to set up enhanced monitoring of the quality and safety of food on and off-campus.
- Run nutrition education programs for students, especially for primary and secondary school students. According to the age group of students, it is recommended to design and carry out various forms of nutrition and health education activities in and out of class.

### Improve the nutrition status of the elderly

Nutrition requirements should respond to the progressive changes in body composition associated with aging. Age-related alterations in physiology and metabolism deserve greater attention in nutrition (18).

#### Promote nutrient screening and dynamic monitoring for the elderly

Development of nutritional screening tools and evaluation systems for the elderly is encouraged. Nutrition screening can start from a pilot program and gradually expand to more than 80% of the elderly in China.

#### Introduce group-specific nutrition improvement measures

Specific measures include medical and health institutions to provide consultation for the individual needs of the elderly, guide hospitals, nursing homes, and other places where the elderly gather to prepare meals in scientific ways, and launch particular nutritional interventions for the seniors underweight.

#### Integrate nutrition management into elderly health management

Specifically, it is recommended to gradually incorporate individual nutrition status into resident health files. Nutrition management is required to be added to the traditional home health care services, thereby promoting the combination of nutrition work and medical care.

### Enhance hospital nutrition care: Nutritious diet can be an important factor in patient treatment and recovery

#### Systematically incorporate nutrition into traditional therapeutic alliances composed of doctors and nurses

Hospital nutrition programs can be started from the pilot. Through talent training and recruitment, the ratio of clinical nutritionists to beds can reach 1:150. Dietitians should be allowed access to multidisciplinary teams for joint patient-specific care (19).

#### Carry out nutritional screening, evaluation, diagnosis, and treatment for inpatients

Health facilities should establish a standardized hospital nutrition care path from screening, evaluation, and diagnosis, to treatment, based on the five-step ladder of nutrition therapy. The effect of therapies should be closely monitored.

Develop and promote the prevention and treatment of diet-related chronic diseases

Nutrition guidelines are suggested to be made for conditions such as hypertension, diabetes, stroke, and cancer. Nutrition assessments are recommended to be carried out for inpatients with diet-related chronic diseases. Nutritional stratification management should be carried out from the hospital and community to the household.

### Implement nutrition interventions in poor areas

#### Incorporate nutrition interventions into the project of health poverty alleviation and provide dietary guidance based on local conditions

To be specific, to launch pilot projects on monitoring the nutrition status, food consumption patterns, intake of major nutrients, and nutrient pollution. Giving classified nutrition guidance and education to residents is recommended. In addition, feasibility studies on agriculture and dietary structures are highly important to optimize local nutrition status.

#### Carry out nutrition interventions for the susceptible and vulnerable population

This initiative requires local health governors to formulate nutrition policies and food standards. Under that framework, pilot programs that provide nutritious meals, improve dining conditions, and monitor nutrition status are advised to be implemented.

Strengthen the monitoring, prevention, and control of foodborne diseases in poor areas, and reduce the prevalence of nutritional deficiencies caused by foodborne diseases.

### Actions on dual track (exercise and diet) health promotion

#### Promote healthy lifestyles

Actions named “three reductions, three health” were carried out in China across regions and age groups.

Three reductions stand for reducing the intake of salt, oil, and sugar. Three health refers to healthy mouth, healthy weight, and healthy bones. Knowledge of healthy dietary patterns can be disseminated through the promotion and application of the Chinese Dietary Guidelines.

#### Provide scientific nutritional support for sportspeople

The support framework includes building a network information service platform for sports people, and a sports nutrition prescription library. In addition, experts can provide precise guidance to specific groups of people to reduce the risk of sports injuries.

Leverage the positive role of exercise in the prevention and rehabilitation of nutrition-related chronic diseases, such as diabetes, obesity, and bone diseases.

## Outlook

The Chinese National Nutrition Plan of Action highlights the urgency to act, guided by an One-Health Approach that identifies six most important and urgent actions to be focused on by 2030.

Regarding the imbalance of nutrition status by population groups, the Nutrition Plan Action proposes operational nutrition intervention recommendations for specific groups, including pre-pregnancy, pregnant women, children, students, the elderly, and patients with chronic diseases.

As summarized by the Global Nutrition Report 2021 (20), Social determinants of nutrition include population density of health workers, sources of drinking water, types and distributions of health facilities, levels of economic development, and population below the poverty line. The heterogeneity of social determinants of nutrition leads to differences in nutritional status among populations in different regions. The Nutrition Plan of Action recognized those heterogeneities and advocated practical actions for participants from all sectors, such as local health professionals and nutrition experts.

Also, the National Nutrition Plan of Action integrated the Nutrition Care Process model (21, 22) that promotes population nutrition status from nutrition assessment, nutrition diagnosis, and nutrition intervention to nutrition monitoring and evaluation. In addition, it recognized the whole nutrition intake life cycle and developed systematic interventions that could facilitate a sustainable development route for the population health. It identified a three-tiered system of intervention from micro-individuals, meso-institutions, to macro-policy. The first is the promotion of health education and nutrition counseling with individuals as the intervention targets. The second is providing support for nutrition-related research and food production with institutions as the targets of intervention. The last tier is to formulate nutrition policies and enhance food quality monitoring with the government as the main body. In this regard, participants from all sectors were mobilized to work together to achieve the “Health China 2030.”

## Strength and limitation

The paper is the first study that articulates the National Nutrition Plan of Action (2017–2030) in China, encompassing analyses of the Action from macro and meso contexts for policy introduction to the practical actions that enable whole sectors to join. The practical experience of China has great potential to be applied to other countries pertaining more to similar urban and rural settings.

Admittedly, the study also has limitations. Data-driven evidence was not presented that could support the introduction of the aforementioned six intervention actions, which may limit the generalization ability of practices. Besides, as the Nutrition Plan of Action has been carried out for 5 years, a dynamic assessment of the progress of each intervention action is of great importance to ensure each target is on track. We will continue to carry out other relevant empirical studies in the future to supplement the limitations of this paper.

## Author contributions

Study conception and design: YT, LC, and XH. Draft manuscript preparation: YT, LC, XH, and XW. Planning and supervision: SL, LW, and HT. All authors contributed to the article and approved the submitted version.

## Funding

This study was supported by funds (92169117) from National Science Foundation of China and the young Top-notch Talent Cultivation Program of Hubei (2021) and Hubei young Top-notch Talent project (2019 and 2021) as well as Hubei outstanding young Science Foundation of Hubei (2020CFA075).

## Conflict of interest

The authors declare that the research was conducted in the absence of any commercial or financial relationships that could be construed as a potential conflict of interest.

## Publisher's note

All claims expressed in this article are solely those of the authors and do not necessarily represent those of their affiliated organizations, or those of the publisher, the editors and the reviewers. Any product that may be evaluated in this article, or claim that may be made by its manufacturer, is not guaranteed or endorsed by the publisher.
